# Adverse Biological Effect of TiO_2_ and Hydroxyapatite Nanoparticles Used in Bone Repair and Replacement

**DOI:** 10.3390/ijms17060798

**Published:** 2016-05-24

**Authors:** Jiangxue Wang, Liting Wang, Yubo Fan

**Affiliations:** 1 Key Laboratory for Biomechanics and Mechanobiology of Ministry of Education, School of Biological Science and Medical Engineering, Beihang University, Beijing 100191, China; wangjiangxue@buaa.edu.cn; 2 National Research Center for Rehabilitation Technical Aids, Beijing 100176, China; wlt6301@sohu.com

**Keywords:** wear debris particles, hydroxyapatite, TiO_2_ nanoparticles, nanotoxicology, cytoxicity, blood protein, inflammatory response, oxidative stress

## Abstract

The adverse biological effect of nanoparticles is an unavoidable scientific problem because of their small size and high surface activity. In this review, we focus on nano-hydroxyapatite and TiO_2_ nanoparticles (NPs) to clarify the potential systemic toxicological effect and cytotoxic response of wear nanoparticles because they are attractive materials for bone implants and are widely investigated to promote the repair and reconstruction of bone. The wear nanoparticles would be prone to binding with proteins to form protein-particle complexes, to interacting with visible components in the blood including erythrocytes, leukocytes, and platelets, and to being phagocytosed by macrophages or fibroblasts to deposit in the local tissue, leading to the formation of fibrous local pseudocapsules. These particles would also be translocated to and disseminated into the main organs such as the lung, liver and spleen via blood circulation. The inflammatory response, oxidative stress, and signaling pathway are elaborated to analyze the potential toxicological mechanism. Inhibition of the oxidative stress response and signaling transduction may be a new therapeutic strategy for wear debris–mediated osteolysis. Developing biomimetic materials with better biocompatibility is our goal for orthopedic implants.

## 1. Introduction

With the advent and rapid development of nanoscience and nanotechnology, every synthetic event occurs at the nanoscale (including fibers, capsules, or particles with at least one dimension from 1 to 100 nm). Especially the engineered nanoparticles (NPs) are massively produced and widely used in the fields of electronics, environment, agriculture, pharmacy, and medicine, *etc.* It is well known that the nanometer regime is the fundamental unit of length over which cells and molecules interact with biological environments. The molecular basic blocks of proteins, nucleic acids, and lipids are materials that possess unique properties at the nanoscale. For example, the width of a DNA strand is approximately 2 nm. The extracellular matrix, providing structural and biochemical support to surrounding cells, has a hierarchical structure with spatial and temporal levels from nanometer to centimeter scale. Now, inspired by the innate nanostructure of biological tissue and biomolecules, many researchers have attempted to fabricate some biomedical nanomaterials with nanoscale surface features to improve biological application in orthopedics [1,2,3,4].

Bone is viewed as a nanofibrous composite with a hierarchical structure composed of organic compounds (mainly collagen) reinforced with inorganic hydroxyapatite (HA). HA crystals are approximately 2 nm thick by 25–50 nm wide, embedded in the holes within the collagen molecule structures and increasing the rigidity of bone. The organization of bone spans three or more orders of magnitude from large ~200 μm osteons with subunits of ~200 nm collagen fibrils to the 20 nm crystallized HA platelets. The specific structure of bone provides mechanical support, metabolic function and protects bone morrow with nutrients in the body. The fracture of bone often occurs because of high force impact and stress, and is also a result of certain medical conditions such as osteoporosis, bone cancer and osteogenesis imperfecta. The broken bone is a lot more than painful and inconvenient, and is sometimes a costly and permanent health problem. According to the National Institutes of Health, approximately 1.5 million hip fractures occur worldwide each year, and this number might increase to 2.6 million by 2025 and 4.5 million by 2050 [5]. The commercial implants, from ceramics to metals to polymers, have some clinical limitations including fatigue, fracture, poor osseointegration, extrusion, and infection. Due to the natural nanostructure of bone, nanotechnology is used to tailor orthopedic implants aimed at helping bone formation and increased integration into the host tissue. To fabricate biomimetic functional bone, many nanomaterials are designed and manufactured, such as titanium dioxide (TiO_2_), HA, ceramics, and nanofibers of polymers. In this review, TiO_2_ and HA are selected as the representative nanomaterials used in orthopedics because they are generally studied as potential biomedical materials, as shown in the following.

## 2. Benefits of TiO_2_ and HA Nanoparticles in Bone Repair

HA with the chemical formula of Ca_10_(PO_4_)_6_(OH)_2_, being the main inorganic constituent of natural bone, has been widely used for biomedical applications owing to good biocompatibility and osteoconductivity [6]. Recently, nano-hydroxyapatite (n-HA) with its small size, high surface area and roughness is more often used than microscale HA for bone substitutes, tissue engineering scaffolds, coatings, and so on [7]. It is playing a more and more important role in bone repair and remodeling [8]. Many studies report that n-HA is used to form a three-dimensional biomimetic composite with chitosan, collagen [9], polymer, and other bioactive molecules [10]. The composite materials of n-HA with natural or synthetic polymer mimic the natural bone’s inorganic and organic phase composition [11]. The n-HA composite scaffolds with appropriate porous structure, biodegradability and mechanical properties can induce osteoblast adhesion, proliferation, and differentiation, increasing their osteoinductivity and osseointegrative capacity [9,12]. The osteoblastic MG63 cells prefer to attach on the gelatin/HA nanocomposites with small-sized apatite crystals, to proliferate, and to secrete alkaline phosphatase (ALP) and osteocalcin (OCN) [13]. Recently, the porous n-HA/collagen scaffold is used to load adriamycin-encapsulated poly(lactic-*co*-glycolic acid) (PLGA) NPs, aiming to develop an osteoconductive material with the ability of inhibiting osteosarcoma [14].

TiO_2_ NPs, which have poor solubility and are chemically inert, are stable in the organism and have good biocompatibility and tissue compatibility. TiO_2_ is considered as a promising bone scaffolding material because it promotes osteoblast adhesion and induces bone formation [15,16,17,18]. Tiainen *et al.* fabricated TiO_2_ scaffolds by themselves and analyzed the bone ingrowth into the scaffold structure after implanting the scaffolds into surgically modified extraction sockets in miniature pigs [18]. Results revealed that the new bone formed in the scaffold pore space by 73.6% ± 11.1%, and the volume of the bone mineral density of the new bone was comparable to that of the native cortical bone. The bone tissue was in direct contact with 50.0 ± 21.5% of the TiO_2_ scaffolds. Webster *et al.* firstly reported that osteoblast cells showed a greater synthesis function of proliferation and osteogenic differentiation on nanophase TiO_2_, ceramics, and HA than conventional ceramics with grain sizes greater than 100 nm [19,20]. TiO_2_ NPs are also used to reinforce the porosity and mechanical properties of polymer scaffolds, aiming to increase bone-forming ability [21,22]. In recent decades, the anodized TiO_2_ nanotubes gain much attention in orthopedic implants due to the nanostructured surface stimulating the growth and mineralization of osteoblasts *in vitro* and inducing bone regeneration and osseointegration *in vivo* [23,24]. The titanium implants with TiO_2_ nanotubes could significantly increase the bone-implant contact and the bone bonding strength [25]. The energy-dispersive X-ray spectroscopy (EDX) mapping exhibited that the strong signals of calcium and phosphorus covered 41.7% of the TiO_2_ nanotube implant surface, but not on the Ti grit-blasted implant surface, indicating the strong osseointegration of TiO_2_ nanotubes [25]. At three weeks after implantation, the titanium implant with 70 nm TiO_2_ nanotubes increased the bone-implant contact by 55.46% ± 9.71%, and the fluorescent labels (xylenol orange and calcein labeled) in the surrounding bone of implants with TiO_2_ nanotubes were more obvious and continuous than those of machined implants [16]. The newly formed bone was observed within the defect site [18] and the high gene expression of ALP, osterix (OSX) and collagen-I were detected in the bone around implants with TiO_2_ nanotubes [16]. The experimental results have clarified that the novel nanoscale HA and TiO_2_ show superior characteristics for bone-related cell adhesion and proliferation due to their distinctive nanoscale features and novel physical properties. On the surface of the titanium disk coated with nanoscale anatase and rutile, human mesenchymal stem cells and osteoblasts are successfully grown and expanded. The bone-specific genes including OSX, ALP, OCN, and bone sialoprotein (BSP) are increased on all nanoscale features *in vitro* [22,26]. In addition, it is reported that n-HA and TiO_2_ nanotubes coated on a titanium surface could prevent bacterial colonization and bacterial biofilm formation on the implant surface [27,28].

However, nanoscale materials usually exhibit unique physical and chemical properties, including magnetic, catalytic, electrical, and mechanical features. Compared to bulk materials, nanomaterials behave very differently. They more easily enter the body and couple with biological entities such as protein, cells, and tissues. There is great concern about the health consequences of nanomaterials due to their extremely small size, high specific surface area, quantum and surface boundary effects, and increased surface activity. The nanostructured surfaces can stimulate cells through initial surface protein adsorption, and activate cell adhesion and proliferation. Evidently, engineered NPs can act as a double-edged sword, either as a platform for osseointegration or as a toxic agent, depending on the context. This has led to increased interest in obtaining an understanding of the biological response to NPs. The potential toxicity of engineered NPs on different cells and tissue has been explored for decades and continues to be investigated. Therefore, the goal of this paper is to analyze the biological data about n-HA and TiO_2_ NPs used as materials of bone implants, to clarify their possible negative effects on the organism and the correlated mechanism.

## 3. Interaction of NPs with Biological Tissue

### 3.1. Interaction with Protein

NPs, when entered into the body, will interact initially with proteins present in biological fluids, such as blood. Protein adsorption onto NPs in biological medium has been taken into consideration as an important factor for the assessment of biological responses to NPs [29,30,31]. Binding to plasma components and forming a nanoparticle-protein corona potentially determine the fate of NPs in the systemic circulation and influence their biological activity [32,33]. In human plasma, a typical protein corona formed on NPs consists of proteins such as serum albumin, immunoglobulins, fibrinogen, and apolipoproteins, *etc.* Nygren *et al.* [34] explored the surface-adsorbed plasma protein and cells on the annealed and acid-oxidized TiO_2_ surface using immunofluorescence technology with short durations of exposure to capillary blood. They detected that serine proteases were the dominating protein adsorbed onto the annealed TiO_2_ and there was a high amount of fibrinogen on the acid-oxidized surface. Platelets could adhere and spread on the annealed surface within 5 s of blood-material contact. When the platelets adhere to the surface they start the signal cascade that eventually leads to cross-linking of fibrin and subsequently to blood clot formation. Using fresh non-anticoagulated human blood, Ekstrand-Hammarström *et al.* [35] detected that TiO_2_ NPs at 50 ng/mL induced strong activation of platelets in the contact system, which could lead to a thrombotic reaction and the generation of bradykinin. Ellingsen [36] examined the reaction of human serum proteins with TiO_2_ and HA surface-adsorbed calcium ions. They identified that the surface of TiO_2_ and HA with calcium binding could selectively take up the albumin, prealbumin and IgG from human serum. The binding of TiO_2_ NPs with human serum albumin involves the van der Waals force and hydrogen bonding formation, and electrostatic interactions might be included [37]. Sousa *et al.* [38] observed that the adsorbed human serum albumin easily exchanged with other albumin molecules or albumin itself. The fibrinogen, γ-globulins, and apolipoprotein could also be adsorbed onto TiO_2_ NPs with the highest affinity than albumin [39,40]. The quantity of adsorbed protein on the large particles is higher than that adsorbed on the small-sized particles [39]. TiO_2_ nanorods evidently adsorb with immunoglobulins IgM and IgG, but TiO_2_ nanotubes mainly bind with fibrinogen. Albumin and apolipoprotein can be adsorbed both on the TiO_2_ nanorods and on the nanotubes [40]. The surface topography and toughness of TiO_2_ nanorods are the only factors that influence the amount of adsorbed fibrinogen [41]. Shen *et al.* [42] studied the adsorption and desorption behavior of fibronectin on the HA surface by molecular dynamics (MD) and steered MD simulations. The research showed that the electrostatic energy played a dominant role in the interaction between the model protein and the HA surface. The bovine serum albumin (BSA) is physically adsorbed through electrostatic attraction between the COOH group of BSA and the calcium ion exposed on the surface of HA [43], which apparently decreases the activation energy and the adsorption heat [44]. The adsorption of BSA, however, onto titanium is tight at low concentrations because the hydration and electrostatic effects are important in the adsorption process [45]. In general, the surface charge and the hydrodynamic diameter of NPs affected by the particle–protein complexes are likely to be what the cell sees in the cell culture medium [46,47]. NP binding, internalization, transport, and even immune response at cellular-level events are highly influenced by the protein corona [48]. In protein-free medium, L929 mouse fibroblasts can uptake more anatase TiO_2_ particles than those in the medium containing human serum albumin. While in the presence of fibrinogen, the particle uptake is lowered by fibroblast cells [39]. Fleischer and Payne [49] reported that in the medium containing BSA, the cellular binding of positively charged BSA–NP complexes was increased, but the cellular binding of negatively charged BSA–NP complexes was inhibited.

### 3.2. Interaction with Blood Cells

It is generally reported that NPs can enter the vascular system intentionally. As a type of active particles, the interaction of NPs with blood cannot be neglected. The previous investigators demonstrated that NPs may have an effect on blood coagulation and hemolysis [50,51,52]. Chen *et al.* [53] evaluated the blood compatibility of n-HA and TiO_2_ both *in vivo* (rats) and *in vitro* (rabbit erythrocytes). The results showed that HA sol obviously prolonged the time of bleeding, clotting and prothrombin in rats after intravenous injection while TiO_2_ sol had no effect. *In vitro*, both NPs’ sol did not cause hemolysis but did induce the aggregation of rabbit red blood cells.

The blood cell is dominated by erythrocytes (99%), with much fewer leukocytes and platelets (1%). The interaction of NPs with erythrocytes has been investigated both *in vivo* and *in vitro*. In a hamster model, Nemmar *et al.* [54] reported the ultrafine carboxylate-polystyrene particles (60 nm) inhibited thrombus formation, while amine-polystyrene significantly enhanced thrombosis and platelet aggregation at 50 μg/mL. The erythrocytes treated with nano-TiO_2_ (20 nm) *in vitro* underwent abnormal sedimentation, hemagglutination and dose-dependent hemolysis, totally differing from those treated with micro-TiO_2_ (200 nm). The ghost cells were observed after exposure to nano-TiO_2_. Han *et al.* [55] investigated the influence of size and surface charge of HA particles on a suspension of red blood cells (RBCs). n-HA NPs induced an obvious aggregation of RBCs, which was different from the HA microparticles. The negatively charged HA NPs inhibited the aggregation of RBCs by heparin modification. Therefore, they concluded that the crucial factor influencing the hemocompatibility of n-HA NPs was the surface charge rather than the particle size [55]. However, the ultra-small TiO_2_ NPs at 1–3 nm showed no cytotoxicity, no oxidative ability and no genotoxicity on human dermal microvascular endothelial cells (HMEC-1) [56]. In early developing zebrafish, TiO_2_ NPs caused mortality and malformations in the form of pericardial edema when injected. The anti-angiogenic effects were also detected both in HMEC-1 cells and in zebrafish embryos, accompanied by a decreased nitric oxide concentration [56]. For the peripheral blood monocytes and lymphocytes, titanium particles induced the production of interleukin (IL)-1β in monocytes, but did not activate lymphocytes because DNA synthesis and IL-2 secretion were unchanged [57]. The toxic effects of TiO_2_ NPs on human umbilical vein endothelial cells (HUVECs) were reported by some scientists. Results showed that TiO_2_ NPs (about 25 nm) at 10, 20, and 40 μg/mL induced higher lactic dehydrogenase (LDH) and superoxide dismutase (SOD) activities in the culture medium (0 μg/mL). When HUVECs were exposed to nano-TiO_2_, IL-6 was significantly released in the medium at 20 and 40 μg/mL and tumor necrosis factor α (TNF-α) was highly released at 5 and 20 μg/mL [58]. The individual or aggregated TiO_2_ NPs internalized into HUVECs strongly inhibited cell proliferation and induced apoptosis by 20% and necrotic death by 60%, even at 5 μg/cm^2^. TiO_2_ NPs provoked the activation of HUVECs through an increase in U937 monocytic adhesion and in the expression of adhesion molecules (E- and P-selectins, ICAM-1, VCAM-1 and PECAM-1), associated with increased reactive oxygen species (ROS) production and nuclear factor (NF)-κB pathway activation [59]. A similar outcome on the cytocompatibility assessment of HA NPs was obtained in THP-1 monocyte–HUVEC endothelial cell co-culture models [60]. HA NPs were taken up by both monocytes and HUVECs, causing the indirect activation and proinflammatory effect of HUVECs through p38/JNK mitogen-activated protein kinases (MAPK) and NF-κB signaling activation. The n-HA resulted in increased total oxidative stress levels, decreased antioxidant capacity, and increased genotoxic effects (increase of sister chromatid exchange, micronuclei, chromosome aberration rates and 8-oxo-2-deoxyguanosine levels) in primary human blood cells in a dose-dependent manner [61]. The potential interaction and adverse effects of NPs on blood cells and cells in the vascular system suggest that NPs could increase the risk of vascular disease and potentially damage the main tissues in the organism through blood circulation.

### 3.3. Local Toxicity

Generally, there are two kinds of tissue responses to particles, the macrophage-mediated response and the lymphocyte-dominated response. In the periprosthetic tissue, Lohmann *et al.* [62] found macrophages containing metal particles and perivascular lymphocytic infiltration and fibrin exudation by histological examination in failed metal-on-metal total hip arthroplasties. By intra-articular injection of TiO_2_ NPs to simulate the release of wear NPs into the joint cavity, Wang *et al.* [63] revealed that the smaller aggregated TiO_2_ NPs penetrated the synovial capillaries and were transported to the heart or lung tissues of rats with blood circulation, and the larger aggregated TiO_2_ particles were deposited in the knee joint, resulting in the injury of the local tissues. For the synovium, the intra-articular TiO_2_ NPs induced fibroblast-like synoviocyte proliferation, lymphocyte and plasma cell infiltration and synovium hypertrophy [63]. The aseptic lesion and enlargement of soft tissue called a pseudotumor is observed by clinicians in the knee/hip joint with prostheses, composed of fibrous connective tissue infiltrated by immunocompetent cells, fibrin deposition and necrosis. Based on the histological changes in periprosthetic tissues, the cause of pseudotumors is thought to be either cytotoxicity of wear particles or a delayed hypersensitivity reaction (DHR or type IV) [64,65]. DHR is not an antibody-mediated response but a type of cell-mediated response with a large number of T cell responses to an external or internal agent.

In the joint, the articular cartilage is avascular and alymphatic and is innervated. The chondrocyte is the only living element of the cartilage. The effect of wear debris on the articular cartilage is investigated through the intra-articular injection of TiO_2_ nanoparticles. The authors [66] detected the decreased thickness of articular cartilage in rats with TiO_2_ NPs at post-exposure days 1, 7, 14, and 30 in a time-dependent manner using the contrast-enhanced high resolution micro-computed tomography. The decreased cartilage volume was detected too. The histopathological changes showed that the chondrocytes had edema with shrunken nuclei in the radial and calcified zone of the cartilage. The cartilage ultrastructure observed by transmission electron microscopy showed the degenerated chondrocytes with condensed chromatin, a dilated endoplasmic reticulum, and rich mitochondria [66].

The wear NPs released from metal-on-metal and metal-on-polyethylene implants circulate in the organism both locally and systemically, penetrate the cell membrane, couple with cellular proteins, and mediate the inflammatory response and expression of cytokines [67]. The macrophages, fibroblasts, and lymphocytes can phagocytose wear debris particles produced from the prosthesis. Titanium and cobalt-chromium particles alter the immune responses when injected into the peritoneal cavity of female mice. At 8 and 12 weeks of injection, titanium and cobalt-chromium particles inhibited cytokine release by lymphocytes (IL-2, IL-4, IFN-γ), proliferation of T and B cells, and immunoglobulin production by B cells. However, these particles are not cytotoxic to murine lymphocytes [68].

The activated macrophage exhibits a spectrum of polarization states, the classically activated M1 phonotype by pro-inflammatory signals (e.g., TNF-α) and the alternatively activated M2 state by anti-inflammatory signals (e.g., IL-4). The proinflammatory signals stimulate the expression of nuclear factor kappa-B ligand (RANKL) on the surface of osteoblasts, and increase the RANKL/OPG (osteoprotegerin) ratio and ROS production by NADPH-oxidase. RANKL interacts with RANK to activate osteoclasts and regulate bone resorption [69,70]. It is also reported that resident macrophages can differentiate into multinucleated functional osteoclasts with exposure to wear particle–mediated cytokine products in an *in vitro* experiment [71].

The inflammatory responses occur and the cytokines are produced including TNF-α, IL-1α, IL-1β, and IL-6. These cytokines stimulate osteoblasts to release soluble cytokines, such as granulocyte-macrophage colony-stimulating factor (GM-CSF), RANKL, IL-6 and prostaglandin E2 (PGE2), or they indirectly stimulate osteoclasts to activate osteolysis [72,73,74]. As a result, the osteogenic activity of osteoblasts is inhibited and predominated by bone resorption at the bone-implant interface. The human synovial cells exhibit a 1.72-fold increase in matrix metalloproteinase (MMP)-2 activity when exposed to a Ti disc and a 3.95-fold increase when exposed to Ti particles [75,76]. Although the cytokine IL-1 only induces a 6.76-fold increase in MMP-2 activity, the combination of Ti particles and inflammatory cytokines induces a 10.54-fold increase of MMP-2 activity. Cytokines released from macrophages and MMPs secreted by fibroblasts can affect the attachment and synthetic activities of osteoblasts and cause the reduction of the bone matrix. The ROS is over-produced in the rat synovial cell line 364 (RSC-364) especially when exposed to 30 and 300 μg/mL anatase TiO_2_ NPs [77,78]. The significantly decreased activities of superoxide dismutase (SOD), intracellular glutathione (GSH) and catalase (CAT) enzymes and the increased lipid peroxidation product (malondiadehyde, MDA) are induced, which means there is oxidative stress and oxidative damage in RSC-364 cells. Choi *et al.* [79] determined the effect of various sizes of Ti particles on the bone-remodeling process both *in vivo* and *in vitro*. By injecting Ti particles with different sizes into the spaces surrounding the Ti alloy pins, they demonstrated that the osteoblast function significantly attenuated with the increased expression of the receptor activator of RANKL, which was a dominant signal for osteoclast recruitment and depended on the size of the Ti particle. Therefore, the chronic proinflammatory response induced by wear particles has a negatively effect on osteoblast function, and decreases bone formation and osseointegration. This maybe a major factor in osteolysis and the subsequent aseptic loosening of arthroplasty implants [80].

### 3.4. Dissemination and Systemic Toxicity

The translocation and accumulation of NPs would occur in the body regardless of the entry routes. There is evidence that NPs can reach and accumulate in the second target organs across body membranes, such as the lung, heart, liver, spleen, and brain. NPs used for bone repair and reconstruction, without exception, would diffuse and redistribute with blood circulation and be entrapped by the reticuloendothelial system (RES). Urban *et al.* [81] firstly reported that metallic wear particles (less than 1 μm) were detected in the paraaortic lymph nodes and further disseminated to the liver and spleen in patients with a failed hip arthroplasty. The submicrometer metal wear particles generated at nonbearing surfaces were identified within macrophages in the liver and spleen of patients with a revised arthroplasty and with primary hip arthroplasty [82]. Using I-125 radiolabeling on n-HA at 80 nm, Sun *et al.* [83] quantitatively analyzed that intravenously administrated n-HA was distributed everywhere in the body with blood circulation, but mainly accumulated in the lung, liver and spleen for over one month.

It is well known that the liver, spleen and kidney are the important metabolic and excretory organs. The special biochemical parameters in serum partially reflect the function of these organs, such as alanine aminotransferase (ALT) and aspartate aminotransferase (AST) for liver function, blood urea nitrogen (BUN) for kidney function and alkaline phosphatase (ALP) for liver function or bone formation–related diseases, and so on. Liu *et al.* [84] found that the n-HAs with a rod shape resulted in acute increases in ALT, AST, BUN and ALP in rabbits after injection by vein. BUN and ALP reached a peak 24 h later, and then decreased rapidly to normal levels. The intraperitoneally injected rod-shaped n-HA (about 20–30 nm in length and 5 nm in width) can be uptaken and disseminate to liver and kidney tissues with the blood circulation; it does not induce the AST, ALT and BUN changes in the serum of rats, but produces apoptosis in the liver cells and renal tubular epithelial cells [85]. However, Wang *et al.* [86] detected that the nano-hydroxyapatite/chitosan (n-HA/CS) composite induced the significant elevation of BUN, CR and total bilirubin (T-Bil) in the serum of SD rats as well as the apoptosis in the liver and kidney tissues with no inflammation and necrosis at eight weeks of exposure by intraperitoneally injection. Sun *et al.* [83] reported that n-HA particles could inhibit the growth of cells and induce cell toxicity in different tissues after intravenous administration, which might be closely related with the residence time of nanoparticles in tissues and the metabolism characteristics of organs.

The translocation of TiO_2_ NPs released from implants is investigated in rats by intra-articular injection [63]. The results demonstrate that the intra-articularly injected TiO_2_ NPs can potentially affect major organs such as the heart, lung and liver, which indicated the circulation of intra-articular NPs from the joint cavity to the system [63]. The slightly pathological change of the heart, lung, and liver is induced by 0.2 mg/kg TiO_2_ and the severe pathological impairment of major organs resulted in the rats after exposure to 2 and 20 mg/kg TiO_2_ suspensions. In the lung tissue, the particle-laden macrophages and the inflammatory cells, such as neutrophils, lymphocytes and eosinophils, are observed in the pulmonary alveoli with thickened alveolar walls. The fatty degeneration of hepatocytes and inflammatory cell infiltration of the portal area prove the injury of the liver. The difficult clearance of TiO_2_ NPs in the lung and liver results in the increased coefficients of organs and the significant upregulation of the serum AST/ALT level, especially in the 20 mg/kg group.

Overall, regardless of whether the NPs were injected or were produced from the prosthesis, once they entered the body, they would circulate everywhere along with the blood circulation and deposit to interact with cells or tissues, leading to the injury of some organs.

### 3.5. Immune Response and Oxidative Stress

As stated above, wear debris particles can activate an inflammatory response locally and systemically. This is mediated by monocytes/macrophages, lymphocytes, synoviocytes, osteoblasts and fibroblasts to secret the products of the proinflammatory cytokines, such as PGE2, IL-6, IL-1β, IL-18, TNF-α [73,87,88], and reactive oxygen intermediates, such as ROS and reactive nitrogen species (RNS). The nanomaterials induce inflammation mainly through oxidative stress, especially for inorganic NPs and metal oxide NPs [89,90]. Both TiO_2_ and HA NPs cause an elevated ROS level and expression of inflammatory transcripts in human oral epithelial cells [91]. Oxidative stress is defined as an imbalance between oxidants and antioxidants and is commonly known to play a role in the formation of a fibrous pseudocapsule around hip implants and to exert negative biological effects [92,93]. Kinov *et al.* [94] detected the high glutathione disulfide (GSSG) and MDA level and low GSH/GSSG ratio in total knee arthroplasties with high wear and osteolysis, suggesting that ROS may be involved in the formation of fibrotic pseudocapsular tissues. In arthrofibrotic tissues, Freeman *et al.* [95] analyzed that the elevated amounts of myeloperoxidase, an enzyme that generates ROS/RNA, downregulated the expression of SOD, glutathione *S*-transferase (GST) and antioxidants and mediated the oxidative DNA and matrix protein modifications (DNA hydroxylation and protein nitrosylation). The over-production of ROS is implicated in the degeneration and necrosis of tissues in fibrous pesudocapsule tissues, and even in diseases. Ding [96] revealed that nano-HA with different sizes could induce pseudo-tubercles in the lung. Additionally, the nanoscale particles with a small size resulted in a vacuolar degeneration of the nephric tubule epithelium after intravenous injection for seven days. In the retrieved hip tissues from total hip anthroplasty patients, the degree of osteolysis shows a correlation with high mobility group protein-B1 (HMGB1), cyclooxygenase-2 (COX2), and 4-hydroxynonenal (4-HNE). This demonstrates that the wear particle–induced oxidative stress mediates bone resorption and osteolysis [97,98].

Many reports state that the structural features of nanoparticles including physical and chemical properties, such as size, shape, crystal phases and surface coating, can exert cytotoxicity through oxidative stress [87,99,100]. Wang and Fan [99] elaborated in detail on the correlation between lung toxicity and pulmonary cell impairment related to TiO_2_ NPs and its unusual physicochemical characteristics, including the size, shape, surface coating, and crystal phases. The ROS mechanism is also elucidated in the toxicity of TiO_2_ NPs. Nano-HA with rod-, spherical-, or needle-shaped crystals can inhibit the growth of primary rat osteoblasts in a dose-dependent manner and induce apoptosis via p53 and the cytochrome C-dependent mitochondrial pathways [101]. Zhao *et al.* [102] demonstrated that nano-HA with smaller specific surface areas induced lower apoptosis, but HA with higher surface area increased the cell-particle interaction and elevated the ROS generation.

The overproduction of ROS is thought of as the best-developed paradigm for the toxicity of NPs [92,93]. With its increase, ROS tends to initiate the significant damage of cell structures and trigger an inflammatory response via the oxidative stress–responsive MAPK, redox-responsive NF-κB and activator protein-1 (AP-1) signaling pathways [103]. Further, wear particle–induced ROS elevation also activates the endoplasmic reticulum (ER) stress markers and promotes apoptosis in osteolysis [104,105]. Yang *et al.* [106] found the phagocytosed wear particles induced the high expression of apoptosis-related markers iNOS, ONOO-, cleaved caspase-3/4/8/9, cytochrome C, glucose regulated protein 78 (GRP78), and growth arrest and DNA damage-inducible gene 153 (GADD153) in macrophages in the periprosthetic interface membrane, proving that the ER stress pathways were the apoptotic pathways of macrophages in the interface membrane of aseptic loosening with the exception of the death receptor pathway and the mitochondrial caspase-dependent pathway. ROS also can improve nanoparticle-induced genotoxicity, which is characterized by chromosomal aberrations, DNA strand breaks, oxidative DNA damage, and mutations [107,108]. In human blood cells, the increases of sister chromatid exchange, micronuclei, chromosome aberration rates and 8-oxo-2-deoxyguanosine levels were caused by HA NPs in a dose-dependent manner [61]. The micronucleus test only showed the type-III foci formation in mouse fibroblasts after exposure to rutile TiO_2_ NPs [109], while there was no increase in DNA damage in human fibroblasts at sub-cytotoxic concentrations [110].

## 4. Conclusions

Depending on good biocompatibility and osteoconductivity, nano-HA and TiO_2_ NPs are generously exploited to increase the repair and reconstruction of bone. However, because of their small size and high surface activity, the adverse biological effects of nanoparticles are an unavoidable scientific problem, which gains much attention from clinicians and scientists. The wear nanoparticles would be prone to adsorb blood proteins to form protein–particle complexes, and further to be phagocytosed by macrophages or fibroblasts and to deposit in the local tissue, leading to delayed hypersensitivity reactions. Meanwhile, the dissemination of nanoparticles into the main organs such as the lung, liver and spleen occurs in the blood circulation system. The inflammatory response, oxidative stress and signaling pathways are elaborated to analyze the toxicological mechanism. Inhibition of redox-related signaling transduction may be a new therapeutic strategy for wear debris–mediated osteolysis. Developing biomimetic materials with better biocompatibility is our goal for orthopedic implants.
